# Cardiac magnetic resonance parameters and ventricular tachycardia recurrence after catheter ablation: a systematic review and meta-analysis

**DOI:** 10.1007/s10840-026-02415-6

**Published:** 2026-08-05

**Authors:** Alexandru Deaconu, Amr Abdin, Mircea Ioan Alexandru Bistriceanu, Silvia Deaconu, Obaida Makdah, Saif Almaghrabi, Guido Matschuck, Cosmin Gabriel Ursu, Sebastian Onciul, Radu Gabriel Vătăsescu

**Affiliations:** 1 Faculty of Medicine, Carol Davila University of Medicine and Pharmacy, Bucharest, 050474 Romania; 2 Clinical Electrophysiology and Pacing Laboratory Emergency Clinical Hospital, Bucharest, 014461 Romania; 3 Internal Medicine Clinic III, Cardiology, Angiology and Intensive Care Medicine, Saarland University Hospital, Homburg/Saar, 66421 Germany; 4 Monza-Ares Hospital, Bucharest, 021967 Romania; 5 Sana Kliniken Niederlausitz, Senftenberg, Germany

**Keywords:** Ventricular tachycardia, Catheter ablation, Cardiac magnetic resonance, Late gadolinium enhancement, Scar characterization, VT recurrence

## Abstract

**Background:**

Cardiac magnetic resonance (CMR) may provide insights into arrhythmogenic substrate in patients undergoing ventricular tachycardia (VT) ablation, but its prognostic value remains uncertain.

**Methods:**

We performed a systematic review and meta-analysis, following PRISMA guidelines, of available studies comparing CMR-derived parameters in patients with and without VT recurrence after ablation, from inception to March 2026. Primary outcomes included functional parameters - left ventricular ejection fraction (LVEF) and left ventricular (LV) mass, while secondary outcomes included ventricular volumes and late gadolinium enhancement (LGE) derived structural characteristics. Random-effects models were applied.

**Results:**

Seven studies including 415 patients were analyzed, with 36.1% experiencing VT recurrence. Lower LVEF was associated with recurrence in the primary analysis (mean difference: − 4.41%; 95% CI: − 8.66 to − 0.16; *p* = 0.044; I²=27.3%), however, this finding was not robust in leave-one-out analysis. No significant associations were observed for LV mass or ventricular volumes. Among structural parameters, transmural, epicardial, or septal LGE were not associated with recurrence.

**Conclusion:**

Available evidence suggests that CMR-derived parameters have limited and inconsistent associations with VT recurrence after catheter ablation. Although reduced LVEF may be associated with recurrence, this finding was not robust in sensitivity analyses, while structural CMR characteristics showed no consistent associations. Larger prospective studies using standardized CMR protocols and time-to-event analyses are needed to better define the prognostic role of CMR.

**Graphical abstract:**

**Figure:**
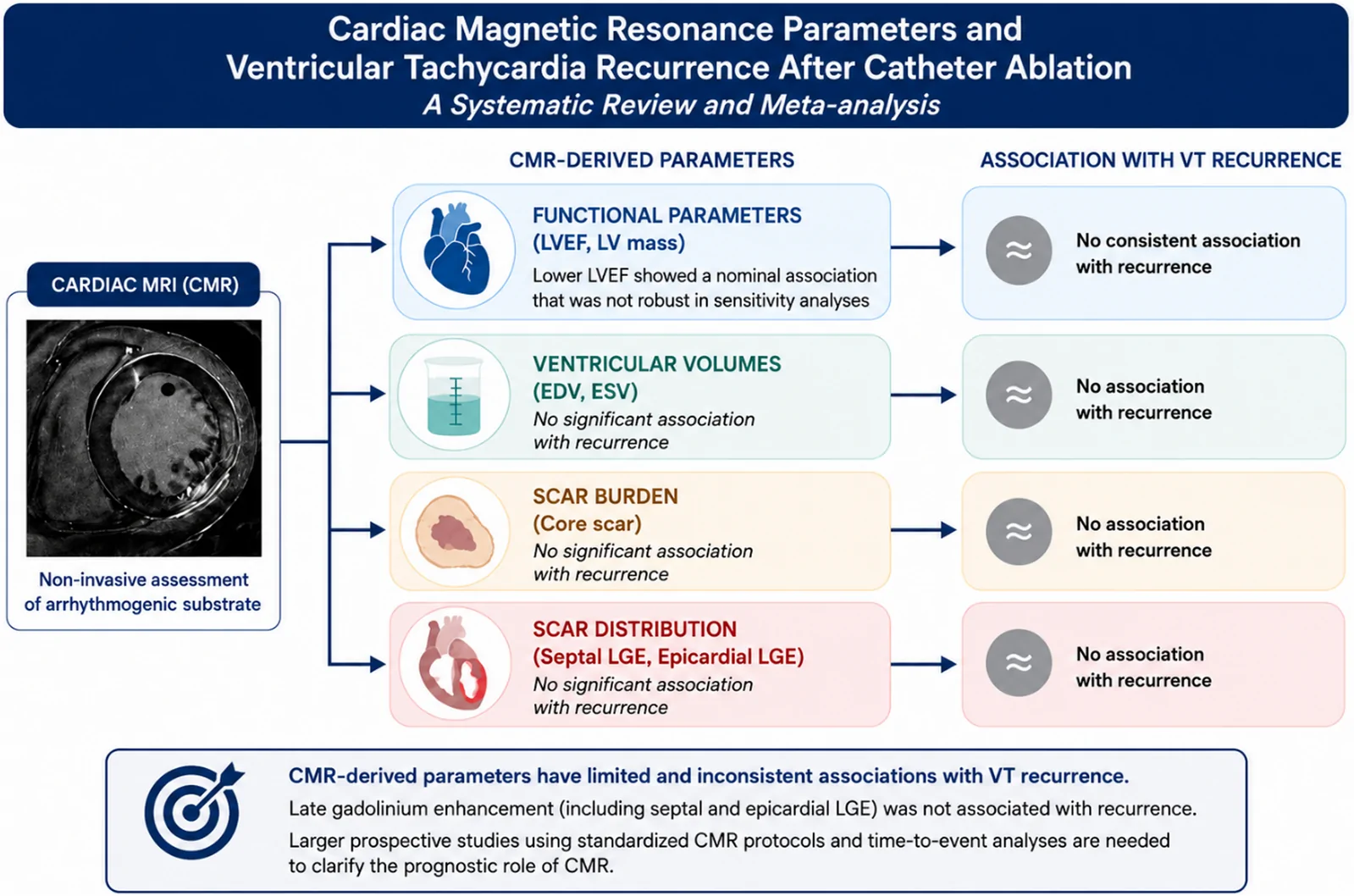

**Supplementary Information:**

The online version contains supplementary material available at https://doi.org/10.1007/s10840-026-02415-6.

## Introduction

Ventricular tachycardia (VT) in patients with structural heart disease remains associated with substantial morbidity and a high risk of recurrence despite advances in catheter ablation strategies. Although ablation reduces arrhythmic burden and the need for implantable cardioverter-defibrillator therapies, recurrence rates remain significant, reflecting the complexity of the underlying arrhythmogenic substrate and variability in procedural approaches and endpoints [1–3]. The substrate of scar-related VT is characterized by heterogeneous myocardial fibrosis with surviving myocardial bundles forming slow-conduction channels that sustain re-entry circuits. Contemporary ablation strategies increasingly rely on substrate modification; however, conventional electroanatomical mapping provides only a limited representation of the three-dimensional architecture of myocardial scar and may fail to identify intramural or epicardial components [4–7]. This limitation is particularly relevant in non-ischemic cardiomyopathies, where fibrosis is often patchy, intramural, or epicardial, contributing to more complex arrhythmogenic circuits and higher recurrence rates following ablation [8].

Cardiac magnetic resonance (CMR) with late gadolinium enhancement (LGE) has emerged as a key modality for non-invasive characterization of the arrhythmogenic substrate. LGE-CMR enables detailed assessment of scar extent, transmurality, and heterogeneity, as well as identification of conducting channels within the scar. In both ischemic and non-ischemic substrates, CMR can detect arrhythmogenic regions not accessible by conventional mapping, supporting more comprehensive substrate delineation and procedural planning [7, 9]. Recent studies have demonstrated associations between CMR-derived parameters - including scar burden, border zone mass, and channel architecture - and ventricular arrhythmias or post-ablation recurrence [10–12]. Despite these advances, the clinical utility of CMR-derived markers in relation to VT recurrence remains incompletely defined. Existing studies are characterized by substantial heterogeneity in imaging protocols, scar quantification methods, and outcome definitions, limiting comparability and generalizability of findings [2, 11]. Furthermore, individual studies often report inconsistent results regarding the prognostic value of specific imaging parameters, including functional indices and scar characteristics [13, 14].

Our systematic review and meta-analysis aimed to assess CMR-derived parameters in patients undergoing VT ablation and to compare imaging features between those with and without VT recurrence, with the goal of identifying consistent imaging markers associated with post-ablation outcomes.

## Methods

The present meta-analysis was performed in accordance with the Cochrane Collaboration and Preferred Reporting Items for Systematic Reviews and Meta-Analysis (PRISMA) statements and has been registered in PROSPERO (CRD420261330592) (Supplementary Table 1) [15].

### Search strategy and data extraction

We systematically searched PubMed, Embase and Cochrane from inception to 30 March 2026 using the terms: “ventricular tachycardia”, “CMR”, “LGE”, and “recurrence”, without any language restrictions. The complete search and PICOTT strategies are available in the Supplemental Material Tables 2 and 3. Case reports, review articles, editorials and conference abstracts were excluded. The references from all included studies, as well as from previous systematic reviews and meta-analyses, were also manually searched for additional studies. Two authors (A.D. and M.I.A.B.) independently extracted the data according to the predefined search criteria and performed the quality assessment.

### Inclusion criteria and endpoints

Studies were included if they enrolled patients undergoing VT ablation with CMR-based substrate characterization and reported recurrence outcomes. Studies lacking CMR evaluation, recurrence stratification, or extractable imaging data were excluded. For the meta-analysis, studies were grouped by the CMR variable of interest, with separate syntheses conducted for functional and structural imaging parameters. For studies reporting both pre- and post-ablation CMR (PAM-VT), only the imaging parameter corresponding to the outcome being analyzed was extracted. Pre-ablation structural variables (e.g., epicardial, septal, or transmural LGE) were synthesized together with comparable pre-procedural measures from other studies, whereas post-ablation parameters (conducting channel number and channel mass) were analyzed separately and were not pooled with pre-ablation substrate characteristics. VT recurrence was analyzed as a binary outcome because most included studies reported cumulative recurrence frequencies rather than comparable time-to-event estimates. Although hazard ratios were available in a minority of studies, differences in adjustment strategies and incomplete reporting precluded a meaningful pooled time-to-event analysis.

The primary outcomes were functional CMR parameters: left ventricular ejection fraction (LVEF) and left ventricular (LV) mass. Secondary outcomes included LV end-diastolic and end-systolic volumes, core scar, presence of septal/transmural/epicardial LGE, number and mass of channels prior to ablation. LV mass and ventricular volumes include only absolute values; index data were not available.

### Study selection and quality assessment

The initial screening was conducted independently by two authors (M.I.A.B. and S.D.), who applied the inclusion/exclusion criteria after removing duplicate studies. The two reviewers independently assessed the full texts for eligibility. Disagreements were resolved through discussion, and if consensus was not achieved, a third reviewer (R.V.) acted as an arbiter. The risk of bias was evaluated by two independent reviewers (A.D. and M.I.A.B.) using the Risk of Bias in Non-Randomized Studies of Interventions (ROBINS-I) assessment (Supplementary Table 4) [16].

### Statistical analysis

For dichotomous variables, odds ratios (OR) and 95% confidence intervals (CI) were calculated using the Hartung-Knapp-Sidik-Jonkman random-effects model. For continuous outcomes, the pooled mean difference (MD) with 95% CI was reported. When continuous variables were reported as median and interquartile range, they were converted to mean and standard deviation using the method proposed by Wan et al.,, applying the appropriate estimator according to the reported summary statistics, to enable quantitative synthesis [17].

Given the variability in VT recurrence definitions, ablation strategies, and CMR protocols, key methodological features were systematically summarized in Supplementary Tables 5 and considered in the assessment of inter-study heterogeneity and interpretation of pooled results. Between-study variance (τ²) was estimated using the restricted maximum-likelihood method, which remains a standard approach in random-effects meta-analyses due to its interpretability and reliable performance across different levels of heterogeneity. Heterogeneity was assessed using τ², I² (percentage of total variability attributable to heterogeneity), and H² (ratio of total to within-study variability). Additionally, Cochran’s Q test was used to statistically evaluate the presence of heterogeneity. Leave-one-out sensitivity analyses were performed for outcomes reported by at least three studies with moderate or high heterogeneity. Publication bias was not formally assessed because fewer than ten studies were available for each outcome. Statistical analyses were performed in RStudio (version 4.5.0).

## Results

### Study and patient characteristics

**Fig. 1:**
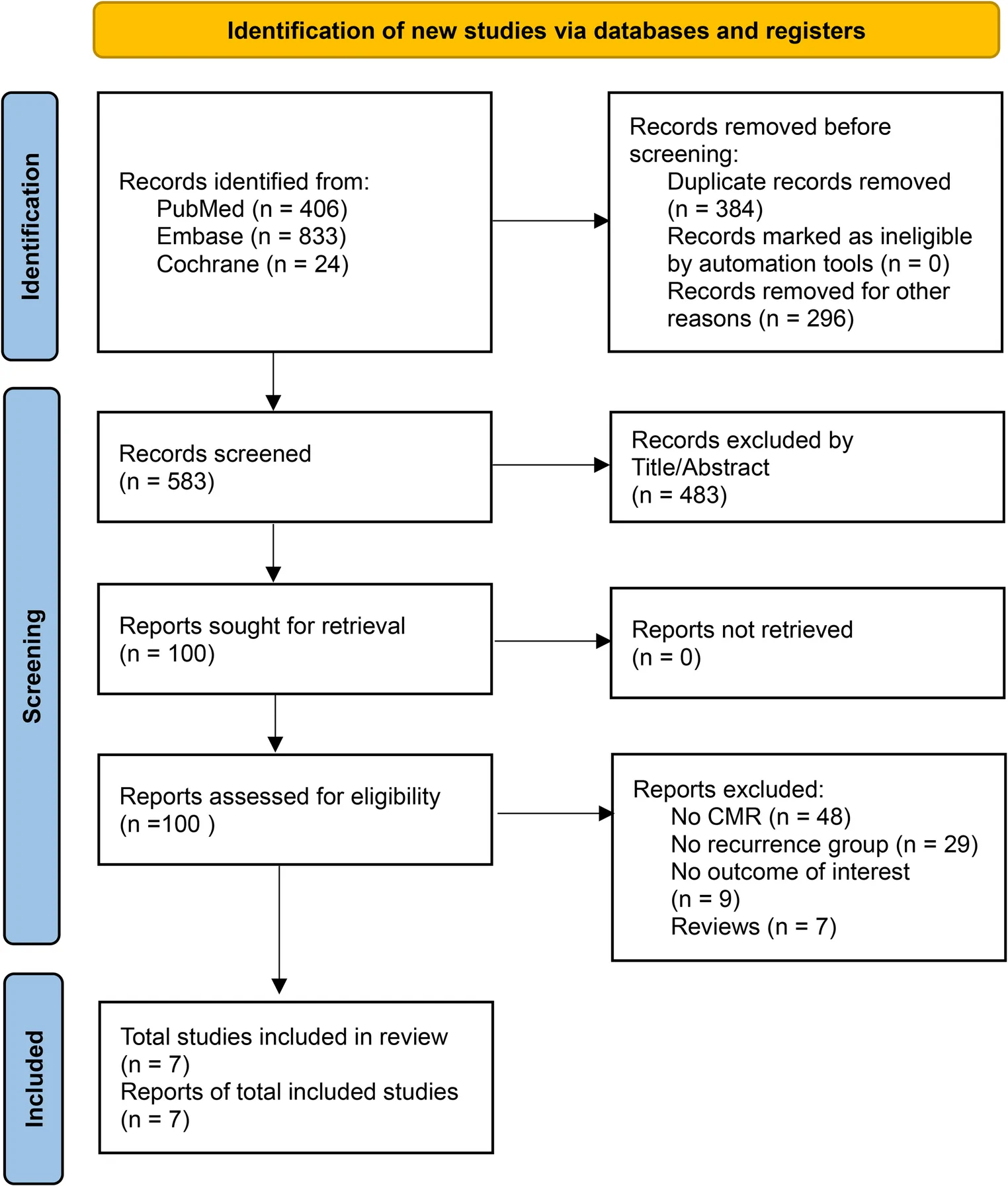
Study selection and flow diagram according to Preferred Reporting Items for Systematic Reviews and Meta-Analyses (PRISMA) guidelines

Our systematic search yielded 1,263 potentially relevant articles (Fig. 1). After removal of duplicates and screening titles and abstracts, 100 studies were assessed for full-text eligibility. Of these, seven observational studies met the inclusion criteria and were included in our meta-analysis, including six studies with pre-procedural CMR and one study with both pre- and post-ablation imaging [13, 14, 18–22]. A total of 415 patients undergoing VT ablation were included, of whom 36.1% experienced VT recurrence. The mean age was 64.4 ± 12.4 years. An ischemic substrate was present in 61% of patients, and the mean baseline LVEF was 36.6 ± 12.6%. The mean follow-up duration was 23.8 months (range 12–32 months). The main characteristics of the included studies are summarized in Table 1. Methodological heterogeneity across the included studies is summarized in Table 2 and detailed in Supplementary Table 5. Although differences existed in cardiomyopathy subtype, CMR acquisition and processing, ablation strategies, recurrence definitions, and follow-up duration, all studies addressed the same clinical question by evaluating the association between CMR-derived structural characteristics and VT recurrence following catheter ablation.

**Table 1:** Baseline characteristics of the patients in the included studies

Study	Country	*N*	Age (years)*	Ischemic substrate, *n* (%)	Baseline LVEF (%) *	VT storm, *n* (%)	Epicardial access, *n* (%)	Mean follow-up (months)
Ávila et al. [18]	Spain	46	68 (9)	46 (100)	36.4 (9.6)	NA	NA	32
Bhagirath et al. [14]	Netherlands	27	72.8 (9.2)	27 (100)	31 (8.1)	NA	NA	31
Njeim et al. [19]	USA	20	59 (17)	8 (40)	42.4 (19.3)	6 (30)	1 (5)	17
Oebel et al. [20]	Germany	147	63.3 (10.5)	63 (42.9)	38.3 (13.1)	111 (76)	38 (26)	20
PAM-VT [21]	Spain	49	65.5 (9.8)	43 (87.7)	34.8 (10.5)	7 (14.2)	6 (12.2)	12
Quinto et al. [13]	Spain	110	63.6 (15)	67 (60.9)	35.6 (13.1)	34 (31)	34 (31)	32
Torri et al. [22]	Germany	16	58 (15)	NA	35.7 (11.6)	NA	NA	12

Age and baseline LVEF are reported as mean and standard deviation

Abbreviations: *SD*, standard deviation; *NA*, not available; *Rec*, recurrence; *LVEF*, left ventricular ejection fraction; *VT*, ventricular tachycardia

**Table 2:** Summary of methodological heterogeneity across included studies

Characteristic	Summary
Cardiomyopathy substrate	ICM: [14, 18] NICM: [22] mixed ICM/NICM: [19, 20] scar-related VT: [13, 21]
Prior ablation	Included in one study only [19]; remaining studies enrolled predominantly first-time ablation patients
VT recurrence definition	ICD therapy or sustained VT [13, 18, 21], documented VT recurrence [19], ICD therapy or VT episodes [14], symptomatic VT episodes [20], VT recurrence at 1 year [22]
Mean follow-up	12–32 months
CMR timing	Pre-ablation; pre- and post-ablation [21]
Scanner field strength	1.5 T [14, 18–20, 22]; 1.5/3 T [13, 21]
Scar quantification	FWHM, SD thresholding, ADAS3D/PSI, conducting-channel analysis
Ablation strategy	Substrate-based, MRI-guided, combined substrate/pacemapping, endocardial ± epicardial
Procedural endpoint	Non-inducibility was targeted in all studies, although the procedural strategies and mapping approaches differed across studies.

Abbreviations: *ADAS3D*, Automatic Detection of Arrhythmogenic Substrate 3D; *FWHM*, Full Width at Half Maximum; *ICD*, implantable cardioverter defibrillators; *ICM*, ischemic cardiomyopathy; *MRI*, magnetic resonance imaging; *NICM*, non-ischemic cardiomyopathy; *PSI*, Pixel Signal Intensity; *SD* thresholding, Standard Deviation thresholding; *VT*, ventricular tachycardia

### Functional CMR parameters

**Fig. 2:**
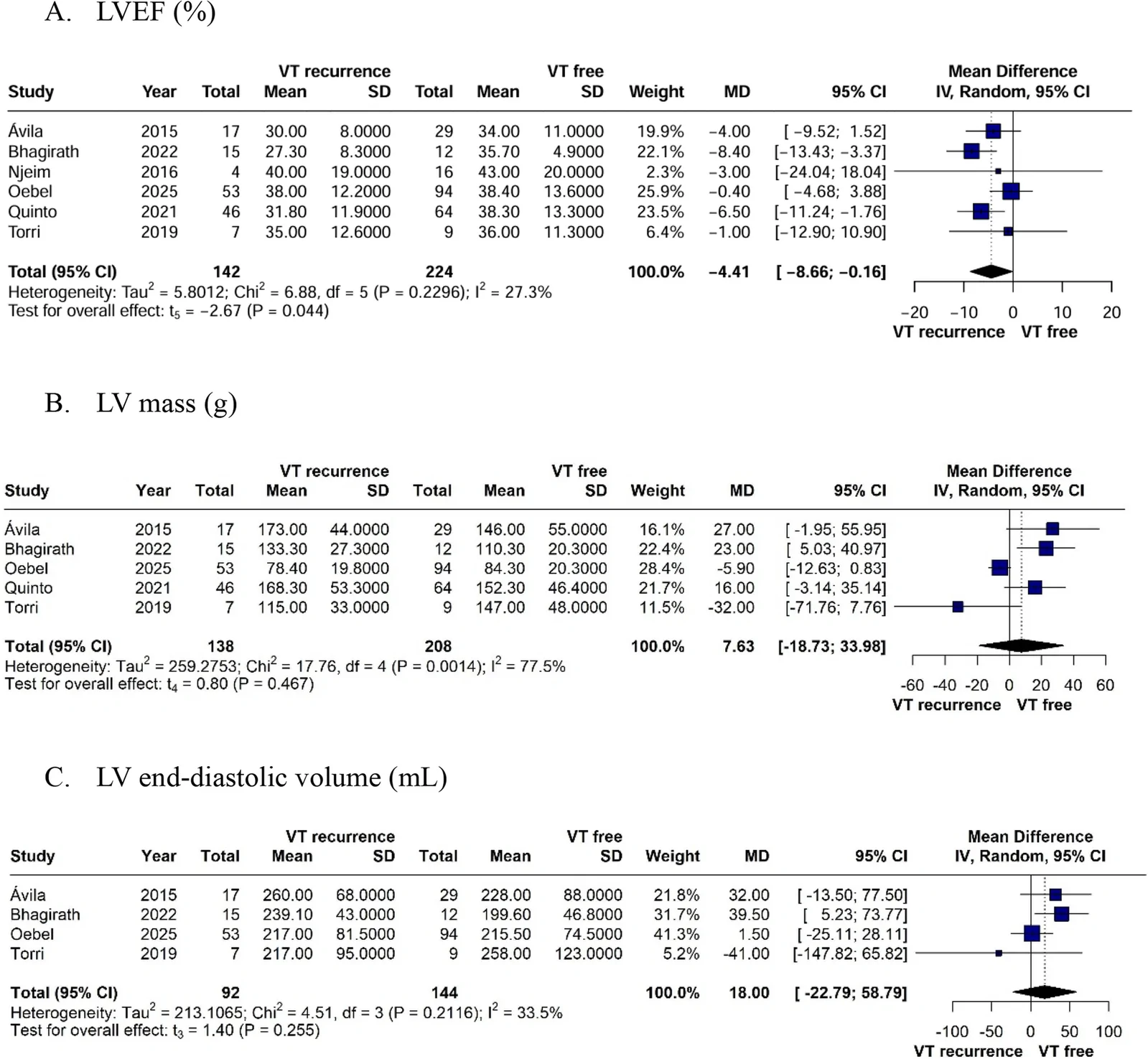

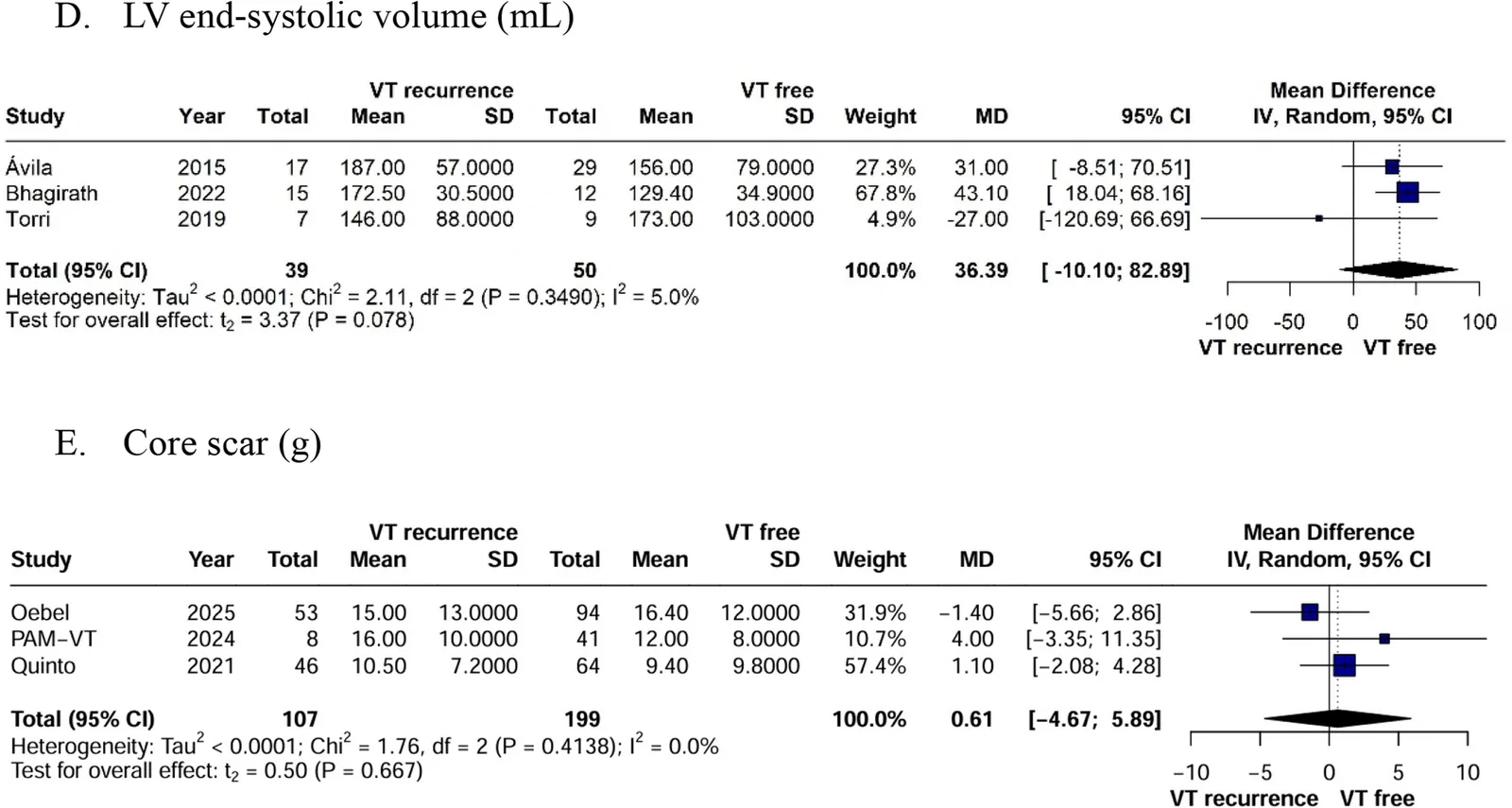
Functional parameters on CMR: **A.** LVEF (%); **B.** LV mass (g); **C.** LV end-diastolic volume (mL); **D.** LV end-systolic volume (mL); **E.** Core scar (g)

Five functional CMR parameters were analyzed across the included studies (Fig. 2). Lower LVEF, reported in 366 patients, was associated with VT recurrence (MD: − 4.41%; 95% CI: − 8.66 to − 0.16; *p* = 0.044), with low to moderate heterogeneity (I² = 27.3%). However, this association was not maintained in a leave-one-out sensitivity analysis after excluding Bhagirath et al. [14] (MD: − 3.28%; 95% CI: − 7.97 to 1.40; *p* = 0.124), with low heterogeneity (Supplementary Fig. 1). LV mass, reported in 346 patients (MD: 7.63 g; 95% CI: − 18.73 to 33.98; *p* = 0.47), LV end-diastolic volume, reported in 236 patients (MD: 18.00 mL; 95% CI: − 22.79 to 58.79; *p* = 0.26), and core scar mass, reported in 306 patients (MD: 0.61 g; 95% CI: − 4.67 to 5.89; *p* = 0.67) were not associated with VT recurrence.

LV end-systolic volume, reported in 89 patients, was also not associated with VT recurrence (MD: 36.39 mL; 95% CI: − 10.10 to 82.89; *p* = 0.078). Heterogeneity was low for LV end-diastolic volume (I² = 33.5%), LV end-systolic volume (I² = 5.0%), and core scar (I² = 0%), but substantial for LV mass (I² = 77.5%). A leave-one-out sensitivity analysis identified Torri et al. [22] as the main contributor to heterogeneity, as its exclusion reduced heterogeneity to 0% without materially altering the direction or significance of the pooled estimate (Supplementary Fig. 2).

### LGE structural parameters

**Fig. 3:**
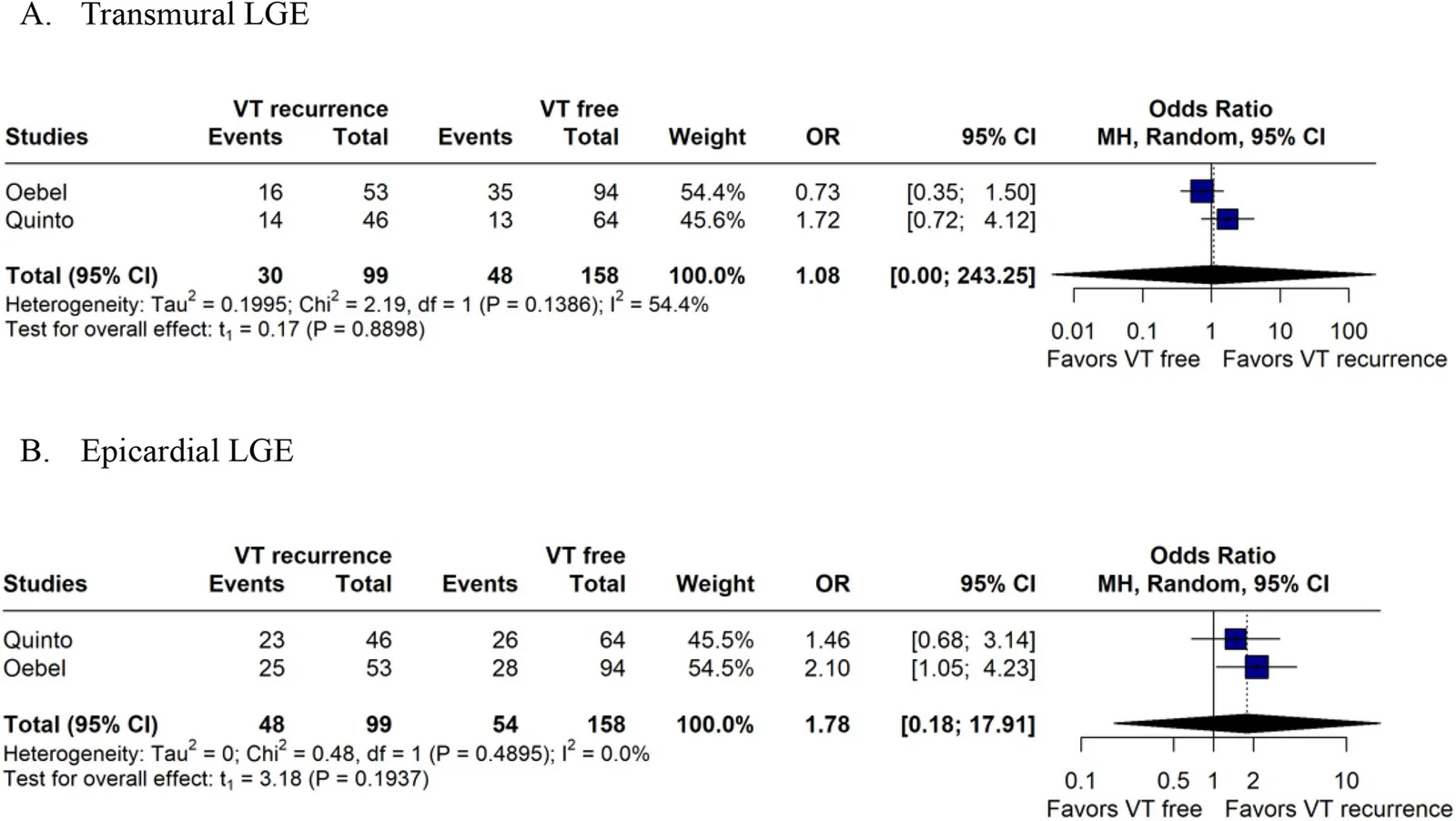

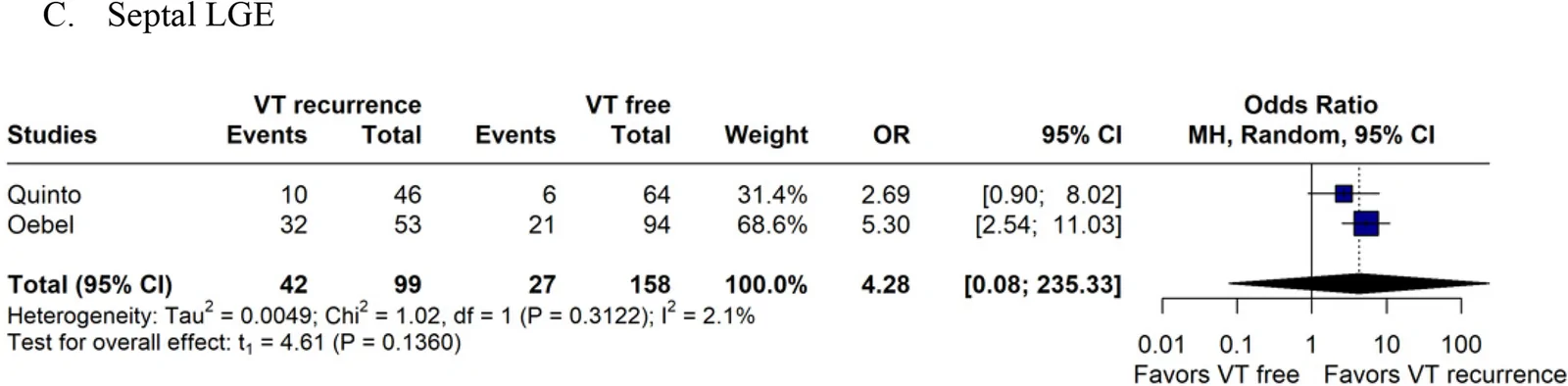
LGE-CMR substrate characterization: **A.** Transmural LGE; **B.** Epicardial LGE; **C.** Septal LGE

LGE-derived structural parameters were analyzed across the included studies, in 257 patients available from only two studies (Fig. 3). The presence of transmural LGE in 78 (30.4%) patients was not associated with VT recurrence (OR: 1.08; 95% CI: 0.00-243.25; *p* = 0.88; I² = 54.4%). The presence of epicardial LGE, in 102 (39.7%) (OR: 1.78; 95% CI: 0.18-17.91; *p* = 0.19; I² = 0%), or septal LGE, in 69 (26.8%) (OR:4.28; 95% CI: 0.08-235.33; *p* = 0.13; I² = 2.1%) were also not associated with VT recurrence.

Additional analyses of advanced substrate characteristics were available in 159 patients, from only two studies. The number of conducting channels and channel mass were not associated with VT recurrence (MD: 0.40; 95% CI: − 4.00 to 4.80; *p* = 0.45, and MD: 0.92 g; 95% CI: − 9.29 to 11.13; *p* = 0.46, respectively (Supplementary Fig. 3). The analyses of the number and mass of conducting channels were derived exclusively from post-ablation CMR in the PAM-VT study and were therefore evaluated separately from pre-procedural substrate characteristics.

## Discussion

In this systematic review and meta-analysis, we compared pre-procedural CMR parameters between patients with and without VT recurrence after catheter ablation to identify imaging characteristics associated with post-procedural VT recurrence. CMR-derived global functional parameters showed limited and inconsistent associations, whereas markers reflecting scar distribution demonstrated potential, albeit preliminary, signals.

Our main findings were:

1. Lower LVEF was associated with recurrence in the primary analysis, but this association was not confirmed in sensitivity analyses.
2. No significant differences were observed in LV mass, ventricular volumes, or core scar mass between groups.
3. Transmural, epicardial, or septal LGE were not associated with recurrence; however, these findings were based on a limited number of studies.
4. Advanced substrate features, including channel number and channel mass, were not associated with recurrence.

### Functional parameters

Global ventricular functional parameters showed inconsistent associations with post-ablation VT recurrence. In the primary analysis, LVEF was lower in patients with recurrence; however, this association was not maintained in sensitivity analyses, suggesting limited robustness. LV volumes and mass did not demonstrate consistent prognostic value across pooled analyses. These findings are partially supported by individual studies included in the analysis. In the cohort analyzed by Bhagirath et al., patients with VT recurrence had significantly lower LVEF and higher LV end-systolic and end-diastolic volumes than those without recurrence [14]. Despite these differences, predictive performance remained modest, and associations were not consistently reproducible across analytical approaches. LV mass was also higher in patients with recurrence in the same study, although its role as an independent marker has not been uniformly confirmed. In the study by Oebel et al., neither LVEF, LV volumes, nor LV mass was associated with VT recurrence following ablation. Quantitative measures of scar burden, including core and border-zone mass, were also not associated with recurrence [20].

Advanced imaging approaches indicate that regional and transmural scar characteristics are more relevant to VT behaviour and recurrence than global scar burden alone. In a translational study using three-dimensional scar reconstruction, total scar volume was not associated with long-term recurrence, whereas transmural scar distribution identified substrate features linked to recurrence risk [7]. Mechanistic studies show that VT circuits are sustained by localized regions of slow conduction within scar tissue, involving complex three-dimensional architectures not captured by global functional indices and dependent on regions of slow and decremental conduction [23]. High-density mapping data indicate that a substantial proportion of critical isthmus regions are defined by rate-dependent functional properties rather than fixed structural scar, highlighting the dynamic nature of the arrhythmogenic substrate [24].

Intramural scar distribution, associated with recurrence in patients with failed ablation procedures, cannot be inferred from LVEF or volumetric parameters alone [19]. Taken together, these observations suggest that global functional parameters, although reflective of overall disease severity, have limited value in relation with VT recurrence after ablation. Detailed characterization of scar architecture, rather than global ventricular function, is required for accurate risk stratification.

### LGE parameters and scar characterization

CMR-LGE enables detailed characterization of the arrhythmogenic substrate, including the presence, distribution, and heterogeneity of scar. The prognostic value of CMR-derived parameters appears to depend on the specific metric assessed, as not all LGE indices carry similar relevance for ventricular arrhythmias or post-ablation recurrence [10, 25]. In the present analysis, quantitative measures of scar burden, including core scar mass and transmural LGE, were not associated with VT recurrence. Similarly, specific patterns of scar distribution, particularly septal and epicardial involvement, were also not associated with recurrence, although reported only in two studies [13, 20]. Neither core nor border-zone mass identified outcomes in the study by Oebel et al., and no CMR-derived parameter demonstrated independent prognostic value [20]. In non-ischemic cardiomyopathy, an increased LGE volume was observed in patients with recurrence, although the relationship between scar burden and electroanatomical substrate remained inconsistent [22].

Scar distribution appears more closely related to arrhythmogenicity. Septal LGE was not associated with recurrence, a finding consistent with the known challenges of septal substrate ablation, including increased wall thickness and intramural circuit location [13]. Epicardial LGE may reflect substrate that is less accessible to endocardial ablation; however, the available data remains limited to determine whether this imaging feature should influence procedural planning or access strategy [21]. These observations support the concept that arrhythmogenic substrate is determined by structural complexity rather than scar extent alone. VT circuits are frequently located within heterogeneous regions containing intramural or subepicardial components that may not be adequately represented by conventional mapping techniques [4, 5]. Integration of CMR-derived scar characterization has been shown to improve identification of critical isthmus regions and guide ablation in complex substrates [26, 27].

High spatial concordance between CMR-defined channels and functional slow-conduction regions supports the ability of imaging to capture the arrhythmogenic substrate, even when it is not fully expressed at baseline [28]. Although stratification by ischemic versus non-ischemic cardiomyopathy could provide additional insight, the available data did not support a robust subgroup analysis in the present meta-analysis. Existing studies suggest that the prognostic value of CMR parameters may differ more by substrate distribution and accessibility than by scar burden alone. In Oebel et al., patients with ischemic cardiomyopathy had higher LV mass (86 vs. 75 g; *p* = 0.005) and LGE core mass (21 vs. 12 g; *p* = 0.001) than the non-ischemic group, whereas border-zone mass was similar (2.9 vs. 2.5 g; *p* = 0.240), and neither core nor border-zone mass was associated with VT recurrence [20]. Vázquez-Calvo et al. reported fewer endocardial channels in non-ischemic substrate than ischemic (44.5% vs. 70.6%; *p* = 0.02) and greater minimal channel depth (3.93 vs. 2.65 mm; *p* = 0.02), while maximal channel depth, not scar or border-zone mass, was associated with recurrence after endocardial ablation [28]. Yamashita et al. showed lower concordance between imaging-defined substrate and local abnormal ventricular activity in patients with non-ischemic cardiomyopathy compared with ischemic cardiomyopathy (72% vs. 90%; *p* < 0.0001), but CMR improved substrate identification compared with multi-detector computed tomography in non-ischemic cardiomyopathy (88% vs. 72%; *p* < 0.001) [29]. Nunes-Ferreira et al. reported similar 36-month efficacy between ischemic and non-ischemic cardiomyopathy after image-guided VT ablation (88.0% vs. 74.1%; *p* = 0.13), despite a much higher use of epicardial ablation in NICM (81.5% vs. 5.3%; *p* < 0.001) [30]. Piers et al. showed that critical isthmus sites in both ischemic and non-ischemic VT were located close to > 75% transmural scar and the core-border-zone transition, supporting the importance of scar architecture rather than total scar extent [31]. Laan et al. found comparable scar quantification between ADAS 3D and inHEART in both substrates, but highlighted that post-processing differences may influence border-zone and channel visualization, particularly in non-ischemic cardiomyopathy [32]. These data are consistent with our findings: core scar mass and global functional parameters were not associated with recurrence, whereas septal and epicardial LGE were associated with VT recurrence.

### Clinical implications and future directions

Variability across studies reflects differences in imaging methodology, patient substrate, and procedural strategy. LGE-CMR provides detailed tissue characterization, but acquisition and post-processing remain non-standardized, limiting reproducibility and cross-study comparability [33]. From a clinical perspective, CMR cannot be used as a standalone tool for risk stratification of VT recurrence. Its role is adjunctive. Pre-procedural imaging may support identification of scar location and transmural distribution, particularly in complex substrates with septal or epicardial involvement [9]. Integration of imaging with electroanatomical mapping improves substrate delineation and procedural targeting, with studies showing lower inducibility and reduced recurrence when CMR-derived scar information is incorporated into ablation strategies [34]. This relationship is further supported by mechanistic data linking CMR-defined channels with critical VT isthmus sites and slow conduction zones [35].

Important substrate features could not be consistently evaluated. Conducting channels, border zone architecture, and scar heterogeneity metrics were not systematically reported across studies. Although emerging data suggest that post-ablation reduction in conducting channels is associated with lower recurrence rates, these parameters were not uniformly available for pooled analysis and remain insufficiently standardized [21, 36]. Advanced approaches, such as artificial-intelligence-based CMR, remain largely investigational and are not represented in current datasets [37]. Clinical outcomes are influenced by factors beyond imaging. Recurrence remains frequent, particularly in advanced or non-ischemic cardiomyopathy, where substrate distribution is often intramural or epicardial and less accessible to conventional ablation [38]. This limits the ability of imaging-derived parameters alone to capture the full complexity of arrhythmogenic substrate.

Future work should focus on standardizing CMR acquisition and analysis, prospectively validating imaging-derived markers, and systematically integrating imaging with electroanatomical mapping. Clear differentiation between ischemic and non-ischemic substrates is required, given their distinct structural and electrophysiological characteristics. Current evidence supports a potential role of advanced scar characterization, but it remains insufficient for routine clinical implementation. Although CMR cannot reliably identify patients at increased risk of VT recurrence, characterization of scar distribution may improve understanding of substrate accessibility and procedural complexity. Current evidence remains insufficient to support the use of specific CMR findings to guide procedural strategy, and prospective validation is required before these imaging features can inform treatment decisions.

## Limitations

This meta-analysis has several limitations that should be considered when interpreting the findings. All included studies were observational and therefore inherently susceptible to residual confounding and selection bias. In addition, the overall number of studies was limited, and for several outcomes only a small number of studies contributed data, potentially statistical power and the precision of pooled estimates. VT recurrence was analyzed as a binary endpoint despite variation in follow-up duration across studies (12–32 months). Because only two studies reported hazard ratios and these were not sufficiently comparable for quantitative synthesis, a pooled time-to-event analysis was not feasible. Consequently, differences in observation time, censoring, mortality, and transplantation could not be accounted for and should be considered when interpreting the pooled estimates. 

Clinical and methodological heterogeneity across the included studies represents another important limitation. Differences in cardiomyopathy subtype, CMR acquisition and post-processing techniques, ablation strategies, recurrence definitions, and follow-up duration may have contributed to variability in the pooled estimates. Although all studies addressed the same overarching clinical question, subgroup analyses according to ischemic and non-ischemic cardiomyopathy were not feasible because of the limited number of studies and overlapping mixed populations. Moreover, our analyses were based on unadjusted aggregate study-level comparisons rather than pooled adjusted prognostic estimates or formal measures of predictive performance. Therefore, the present findings should be interpreted as associations between CMR-derived parameters and VT recurrence rather than evidence of independent predictive ability. Post-ablation CMR analyses were available from a single study and inherently included only patients who survived the procedure and underwent follow-up imaging, introducing potential selection bias.

## Conclusion

In this systematic review and meta-analysis of observational studies, currently available evidence does not support robust associations between individual CMR-derived parameters and VT recurrence after catheter ablation. Although a lower LVEF showed a directional signal across studies, CMR data remains limited by small sample sizes, methodological heterogeneity, and imprecise effect estimates. Future prospective studies using standardized CMR acquisition, scar characterization, and outcome definitions are needed to clarify the role of CMR in VT recurrence risk assessment.

## Supplementary Information

Below is the link to the electronic supplementary material.

Supplementary Material 1

## Data Availability

No datasets were generated or analysed during the current study.

## Abbreviations

CI: Confidence interval
CMR: Cardiac magnetic resonance
LGE: Late gadolinium enhancement
LV: Left ventricular
LVEF: Left ventricular ejection fraction
MD: Mean difference
OR: Odds ratio
PRISMA: Preferred Reporting Items for Systematic Reviews and Meta-Analyses
ROBINS-I: Risk of Bias in Non-Randomized Studies of Interventions
VT: Ventricular tachycardia
