# Supplementary material for: Cardiac magnetic resonance parameters and ventricular tachycardia recurrence after catheter ablation: a systematic review and meta-analysis
Source: J Interv Card Electrophysiol. 2026 Aug 5;69(9):3231–42. doi: 10.1007/s10840-026-02415-6 (PMC13769757; doi:10.1007/s10840-026-02415-6)
Supplement: Supplementary file 1 [file 10840_2026_2415_MOESM1_ESM.docx]

|  | **Item #** | **Checklist item** | **Location where item is reported** |
| --- | --- | --- | --- |
| **TITLE** | | |  |
| Title | 1 | Identify the report as a systematic review. | Lines 1-2 |
| **ABSTRACT** | | |  |
| Abstract | 2 | See the PRISMA 2020 for Abstracts checklist. | Line 45 |
| **INTRODUCTION** | | |  |
| Rationale | 3 | Describe the rationale for the review in the context of existing knowledge. | Lines 111-116 |
| Objectives | 4 | Provide an explicit statement of the objective(s) or question(s) the review addresses. | Lines 117-119 |
| **METHODS** | | |  |
| Eligibility criteria | 5 | Specify the inclusion and exclusion criteria for the review and how studies were grouped for the syntheses. | Lines 139-142 |
| Information sources | 6 | Specify all databases, registers, websites, organisations, reference lists and other sources searched or consulted to identify studies. Specify the date when each source was last searched or consulted. | Lines 129-131 |
| Search strategy | 7 | Present the full search strategies for all databases, registers and websites, including any filters and limits used. | Supplementary Table 3 |
| Selection process | 8 | Specify the methods used to decide whether a study met the inclusion criteria of the review, including how many reviewers screened each record and each report retrieved, whether they worked independently, and if applicable, details of automation tools used in the process. | Lines 150-152 |
| Data collection process | 9 | Specify the methods used to collect data from reports, including how many reviewers collected data from each report, whether they worked independently, any processes for obtaining or confirming data from study investigators, and if applicable, details of automation tools used in the process. | Lines 134-135 |
| Data items | 10a | List and define all outcomes for which data were sought. Specify whether all results that were compatible with each outcome domain in each study were sought (e.g. for all measures, time points, analyses), and if not, the methods used to decide which results to collect. | Lines 144-146 |
|  | 10b | List and define all other variables for which data were sought (e.g. participant and intervention characteristics, funding sources). Describe any assumptions made about any missing or unclear information. | Not applicable |
| Study risk of bias assessment | 11 | Specify the methods used to assess risk of bias in the included studies, including details of the tool(s) used, how many reviewers assessed each study and whether they worked independently, and if applicable, details of automation tools used in the process. | Lines 153-155 |
| Effect measures | 12 | Specify for each outcome the effect measure(s) (e.g. risk ratio, mean difference) used in the synthesis or presentation of results. | Lines 159-161 |
| Synthesis methods | 13a | Describe the processes used to decide which studies were eligible for each synthesis (e.g. tabulating the study intervention characteristics and comparing against the planned groups for each synthesis (item #5)). | Lines 139-142 |
|  | 13b | Describe any methods required to prepare the data for presentation or synthesis, such as handling of missing summary statistics, or data conversions. | Lines 159-170 |
|  | 13c | Describe any methods used to tabulate or visually display results of individual studies and syntheses. | Lines 159-170 |
|  | 13d | Describe any methods used to synthesize results and provide a rationale for the choice(s). If meta-analysis was performed, describe the model(s), method(s) to identify the presence and extent of statistical heterogeneity, and software package(s) used. | Lines 159-170 |
|  | 13e | Describe any methods used to explore possible causes of heterogeneity among study results (e.g. subgroup analysis, meta-regression). | Lines 159-170 |
|  | 13f | Describe any sensitivity analyses conducted to assess robustness of the synthesized results. | Lines 159-170 |
| Reporting bias assessment | 14 | Describe any methods used to assess risk of bias due to missing results in a synthesis (arising from reporting biases). | Lines 153-155 |
| Certainty assessment | 15 | Describe any methods used to assess certainty (or confidence) in the body of evidence for an outcome. | Not applicable |
| **RESULTS** | | |  |
| Study selection | 16a | Describe the results of the search and selection process, from the number of records identified in the search to the number of studies included in the review, ideally using a flow diagram. | Lines 176-179, Figure 1 |
|  | 16b | Cite studies that might appear to meet the inclusion criteria, but which were excluded, and explain why they were excluded. | Figure 1 |
| Study characteristics | 17 | Cite each included study and present its characteristics. | Lines 179-183 |
| Risk of bias in studies | 18 | Present assessments of risk of bias for each included study. | Supplementary Table 4 |
| Results of individual studies | 19 | For all outcomes, present, for each study: (a) summary statistics for each group (where appropriate) and (b) an effect estimate and its precision (e.g. confidence/credible interval), ideally using structured tables or plots. | Lines 246-293 |
| Results of syntheses | 20a | For each synthesis, briefly summarise the characteristics and risk of bias among contributing studies. | Lines 246-293 |
|  | 20b | Present results of all statistical syntheses conducted. If meta-analysis was done, present for each the summary estimate and its precision (e.g. confidence/credible interval) and measures of statistical heterogeneity. If comparing groups, describe the direction of the effect. | Lines 246-293 |
|  | 20c | Present results of all investigations of possible causes of heterogeneity among study results. | Lines 246-293 |
|  | 20d | Present results of all sensitivity analyses conducted to assess the robustness of the synthesized results. | Lines 246-293 |
| Reporting biases | 21 | Present assessments of risk of bias due to missing results (arising from reporting biases) for each synthesis assessed. | Supplementary Table 4 |
| Certainty of evidence | 22 | Present assessments of certainty (or confidence) in the body of evidence for each outcome assessed. | Not applicable |
| **DISCUSSION** | | |  |
| Discussion | 23a | Provide a general interpretation of the results in the context of other evidence. | Lines 296-309 |
|  | 23b | Discuss any limitations of the evidence included in the review. | Lines 434-444 |
|  | 23c | Discuss any limitations of the review processes used. | Lines 434-444 |
|  | 23d | Discuss implications of the results for practice, policy, and future research. | Lines 310-433 |
| **OTHER INFORMATION** | | |  |
| Registration and protocol | 24a | Provide registration information for the review, including register name and registration number, or state that the review was not registered. | Line 125 |
|  | 24b | Indicate where the review protocol can be accessed, or state that a protocol was not prepared. |  |
|  | 24c | Describe and explain any amendments to information provided at registration or in the protocol. |  |
| Support | 25 | Describe sources of financial or non-financial support for the review, and the role of the funders or sponsors in the review. | Lines 476-478 |
| Competing interests | 26 | Declare any competing interests of review authors. | Lines 474-475 |
| Availability of data, code and other materials | 27 | Report which of the following are publicly available and where they can be found: template data collection forms; data extracted from included studies; data used for all analyses; analytic code; any other materials used in the review. | Supplementary material |

Supplementary Table 1. PRISMA checklist

| *P* | Patients with structural heart disease and non-ischemic cardiomyopathy undergoing VT ablation and cardiac magnetic resonance |
| --- | --- |
| *I* | Patients with recurrence of ventricular tachycardia |
| *C* | Patients without recurrence of ventricular arrhythmias |
| *O* | CMR parameters – pre or post ablation |
| *T* | Randomized controlled trials and observational cohort studies |
| *T* | No time restriction on follow-up |

Supplementary Table 2. PICOTT strategy

| Database | Search strategy |
| --- | --- |
| PubMed | (ventricular tachycardia AND ablation) AND ("late gadolinium enhancement" OR LGE OR "scar" OR "border zone" OR "fibrosis") AND (recurrence OR "arrhythmia recurrence") |
| Embase | ('ventricular tachycardia'/exp OR 'ventricular tachycardia') AND ('catheter ablation'/exp OR ablation) AND ('magnetic resonance imaging'/exp OR mri OR cmr OR 'late gadolinium enhancement') AND (recurrence OR prognosis OR outcome) |
| Cochrane | ventricular tachycardia AND ablation AND (MRI OR CMR OR LGE) |

Supplementary Table 3. Search strategies

Supplementary Table 4. Risk of bias – ROBINS-I

| Study | Population | VT recurrence definition | VT ablation strategy | CMR timing | CMR protocol |
| --- | --- | --- | --- | --- | --- |
| Ávila, 2015 | Ischemic cardiomyopathy  (prior myocardial infarction) | ICD therapy or sustained VT | Endocardial substrate ablation (LPs/CCs elimination) | Pre-ablation | 1.5T LGE-CMR;  FWHM + SD thresholds;  3D SI mapping |
| Bhagirath, 2022 | Ischemic cardiomyopathy | ICD therapy or VT episodes | Combined (substrate + pacemapping), non-inducibility endpoint | Pre-ablation | 1.5T LGE-CMR; ADAS3D; PSI mapping; corridor/ interface analysis |
| Njeim, 2016 | Structural heart disease (ICM + NICM), failed prior ablation | Documented VT recurrence (ICD/clinical) | MRI-guided tailored (endo/epi/combined), non-inducibility endpoint | Pre-ablation | 1.5T DE-MRI; FWHM; scar classified (endo/epi/intramural) |
| Oebel, 2025 | Structural heart disease (ICM + NICM) | Symptomatic VT / ICD therapy / sustained VT (no blanking) | Substrate-based (endo ± epi), non-inducibility endpoint | Pre-ablation | 1.5T LGE-CMR; FWHM; core (>60%) + border zone (40-60%) |
| PAM-VT, 2024 | Scar-related VT (predominantly ICM) | VT >30s or ICD therapy | Substrate-based (EAM + CMR-guided), non-inducibility | Pre + Post ablation | LGE-CMR (1.5T/3T); PSI mapping; scar and conducting channel analysis |
| Quinto, 2021 | Scar-related VT (ICM + NICM) | VT >30s or ICD therapy | Substrate-based (scar dechanneling), LP abolition + non-inducibility | Pre-ablation | LGE-CMR (1.5T/3T); PSI mapping; scar and conducting channel characterization |
| Torri, 2019 | Non-ischemic dilated cardiomyopathy | VT recurrence at 1 yr (ICD/Holter) | RF ablation (VT/VA), non-inducibility endpoint | Pre-ablation | 1.5T LGE-CMR; FWHM quantification; LGE volume assessment |

Supplementary Table 5. Methodological heterogeneity across included studies: population, VT recurrence definitions, ablation strategies, and CMR acquisition protocols

Supplementary Figure 1. Leave-one-out analysis for LVEF – after excluding Bhagirath et al.

A. Excluding Oebel et al.

B. Excluding Torri et al.

Supplementary Figure 2. Leave-one-out analysis for LV mass.

A. Number of channels

B. Channels mass (g)

Supplementary Figure 3. Conduction channel analysis: A. Number of channels; B. Channel mass.
